# The lipid environment determines the activity of the *Escherichia coli* ammonium transporter AmtB

**DOI:** 10.1096/fj.201800782R

**Published:** 2018-09-13

**Authors:** Gaëtan Dias Mirandela, Giulia Tamburrino, Paul A. Hoskisson, Ulrich Zachariae, Arnaud Javelle

**Affiliations:** * Strathclyde Institute of Pharmacy and Biomedical Sciences, University of Strathclyde, Glasgow, United Kingdom;; † Computational Biology, School of Life Sciences, University of Dundee, Dundee, United Kingdom;; ‡ Physics, School of Science and Engineering, University of Dundee, Dundee, United Kingdom

**Keywords:** Amt/Mep/Rh, protein–lipids interaction, molecular dynamics simulation, SSME

## Abstract

The movement of ammonium across biologic membranes is a fundamental process in all living organisms and is mediated by the ubiquitous ammonium transporter/methylammonium permease/rhesus protein (Amt/Mep/Rh) family of transporters. Recent structural analysis and coupled mass spectrometry studies have shown that the *Escherichia coli* ammonium transporter AmtB specifically binds 1-palmitoyl-2-oleoyl phosphatidylglycerol (POPG). Upon POPG binding, several residues of AmtB undergo a small conformational change, which stabilizes the protein against unfolding. However, no studies have so far been conducted, to our knowledge, to explore whether POPG binding to AmtB has functional consequences. Here, we used an *in vitro* experimental assay with purified components, together with molecular dynamics simulations, to characterize the relation between POPG binding and AmtB activity. We show that the AmtB activity is electrogenic. Our results indicate that the activity, at the molecular level, of Amt in archaebacteria and eubacteria may differ. We also show that POPG is an important cofactor for AmtB activity and that, in the absence of POPG, AmtB cannot complete the full translocation cycle. Furthermore, our simulations reveal previously undiscovered POPG binding sites on the intracellular side of the lipid bilayer between the AmtB subunits. Possible molecular mechanisms explaining the functional role of POPG are discussed.—Mirandela, G. D., Tamburrino, G., Hoskisson, P. A., Zachariae, U., Javelle, A. The lipid environment determines the activity of the *Escherichia coli* ammonium transporter AmtB.

Ammonium is a vital source of nitrogen for bacteria, fungi, and plants and a toxic metabolic waste product for animals (1). Hence, ammonium transport across biologic membranes is a process of fundamental importance in all living organisms. In 1994, the first genes encoding ammonium transporters were identified in *Saccharomyces cerevisiae* [methylammonium permease (*Mep*)] (2) and *Arabidopsis thaliana* [ammonium transporter (*Amt*)] (3). Later, it was shown that the rhesus protein (Rh) is an ortholog of Amt in vertebrates (4) and, remarkably, that yeast *Mep* mutants can be complemented with the human Rh glycoprotein (5), demonstrating that the Rh protein is a functional ammonium transporter. Following those seminal findings, members of the Amt/Mep/Rh protein family were identified in almost all sequenced organisms, forming a unique and highly specific family of ammonium transporters (6, 7).

The functional context of Amt/Mep and Rh transporters is highly diverse: bacteria, fungi, and plants use Amt/Mep proteins to scavenge ammonium from their environments for biosynthetic assimilation, whereas mammals use the Rh proteins for ammonium detoxification in erythrocytes and kidney and liver tissues (1, 8, 9). Hence, members of the Amt/Mep/Rh family of proteins are associated with various fundamental biologic processes. In fungi, the dimorphic transition from yeast to filamentation is often related to the virulence of pathogenic species, such as *Candida albicans* (10), *Histoplasma capsulatum* (11), and *Cryptococcus neoformans* (12). Fungi possess multiple Mep proteins, and it has been shown that, in *S. cerevisiae* (13), the plant pathogens *Ustilago maydis* (14, 15), *Fusarium fujikuroi* (16), and the human pathogen *C. albicans* (17), the Mep2 transporters have a key role in the switch to filamentous growth.

In humans, Rh mutations are associated with numerous pathologies. RhAG mutations in red blood cells have been linked to recessive rhesus protein deficiency (18) and overhydrated hereditary stomatocytosis (19), a rare, dominant-inherited hemolytic anemia. In mouse kidneys, RhCG mutations impair ammonium homeostasis and are associated with distal renal tubular acidosis and male infertility (20). Finally, RhCG has been identified as a candidate gene for early onset major depressive disorder (21).

The *Escherichia coli* ammonium transporter AmtB is the most widely studied model system to investigate ammonium uptake in the ubiquitous Amt/Mep/Rh protein family (22). AmtB is well characterized structurally, with >20 high-resolution structures reported in the Protein Data Bank (PDB; Research Collaboratory for Structural Bioinformatics, *https://www.rcsb.org/*) to date. Despite this wealth of structural information, the ammonium transport mechanism has not yet been unraveled from those crystal structures because all the structures show a very similar conformation reflecting the inward-facing state of the protein, irrespective of the presence or absence of ammonium. Recently, mass spectrometry analysis coupled with structural studies defined 8 specific binding sites for the lipid 1-palmitoyl-2-oleoyl phosphatidylglycerol l (POPG) head group in AmtB, which increase protein stability (23). The X-ray structure of AmtB with bound POPG reveals distinct conformational changes, which reposition some of the protein residues that interact with lipids. More recently, it has been shown that POPG can allosterically regulate the interaction between AmtB and the signal transduction protein GlnK (24). In spite of those findings, a direct functional role for POPG on the transport of ammonium by AmtB has remained unclear.

Here, we couple an *in vitro* assay, based on protein reconstitution in liposomes and solid supported membrane electrophysiology (SSME) measurements with molecular dynamics (MD) simulations to illuminate the effect of lipid composition on AmtB activity. Our results indicate that the function of Amt in archaebacterial and eubacteria differs. We also show that POPG is an essential cofactor for AmtB activity and that, in the absence of POPG, AmtB cannot complete the full translocation cycle. To our knowledge, this is the first report highlighting the functional importance of specific lipids for AmtB activity and demonstrating that the high AmtB selectivity for POPG lipids is not only important for protein stability but also for the translocation cycle.

## MATERIALS AND METHODS

### Protein purification

AmtB(His_6_), cloned into the pET22b vector, was overproduced and purified as previously described (9), except that 0.03% of *n*-dodecyl-β-d-maltoside (DDM) was used, instead of 0.09% *N*,*N*-dimethyldodecylamine-*N*-oxide in the final size-exclusion chromatography (SEC) buffer (Tris/HCl 50 mM, pH7.8, NaCl 100 mM, 0.03% DDM) (25). AmtB was kept in the SEC buffer at 4°C for subsequent characterization and insertion into proteoliposomes.

### Reconstitution in liposomes

All lipids (Avanti Polar Lipids, Alabaster, AL, USA) were dried under nitrogen flow and resuspended at 5 mg/ml in nonactivating (NA) buffer. The multilamellar liposomes were subsequently extruded 13 times with the miniextruder (Avanti Polar Lipids) mounted with a 0.1-µm filter pore. To facilitate the insertion of AmtB into liposomes, 1 µl Triton X-100 at 25% was sequentially added to 500 µl of liposomes, and the absorbance at 400, 500, 550, and 600 nm was measured to determine the saturation and solubilization constants. The liposomes were incubated for 5 min at room temperature. AmtB, stabilized in 0.03% DDM, was added at a lipid-to-protein ratio (LPR) of 5:1, 10:1, or 50:1 (w/w), and the mixture left for 30 min at room temperature. Three subsequent incubations with prewashed SM-2 Biobeads (Bio-Rad Laboratories, Hercules, CA, USA) at a beads-to-detergent ratio (w/w) of 20 were performed to ensure detergent removal and AmtB insertion. The average diameter of the liposomes/proteoliposomes was determined by dynamic light scattering (DLS) with a Zetasizer Nano ZS (Malvern Instruments, Malvern, United Kingdom) (Supplemental Fig. S1). Proteoliposomes were divided into 100-µl aliquots and frozen at −80°C.

### AmtB orientation

**Figure 1:**
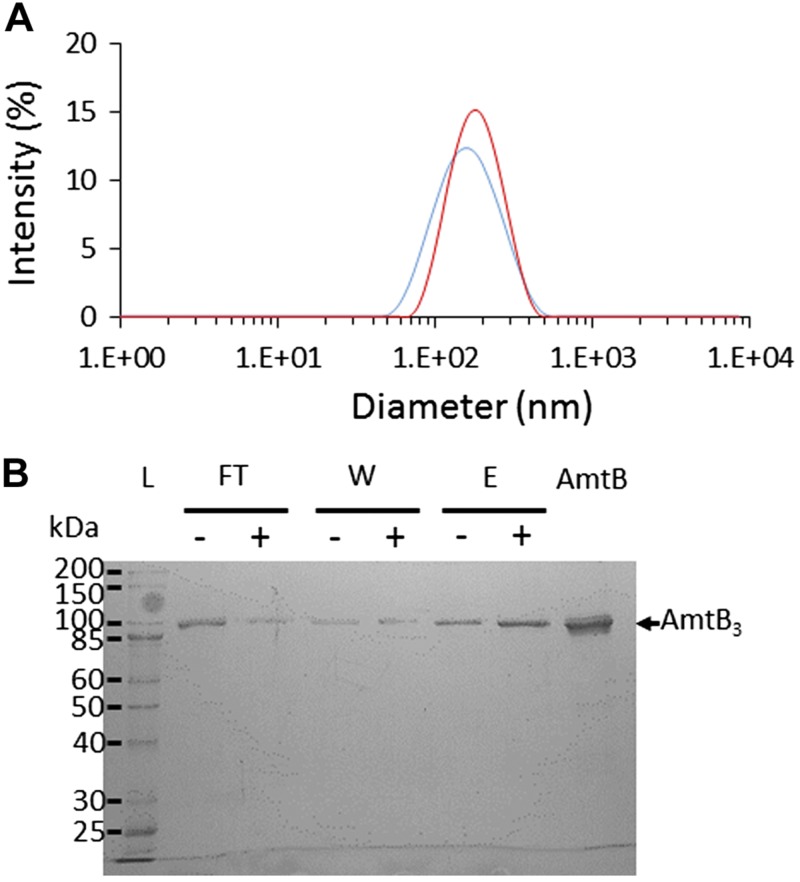
AmtB purification and reconstitution into liposomes (condition 1, table 1). *A*) DLS analysis of the empty liposomes (blue) and proteo-liposomes (red). *B*) SDS-PAGE Coomassie Blue–stained gel of the liposomes purified by IMAC after DDM treatment (+) or in absence of DDM (−). AmtB, 5 μg of pure AmtB used for the reconstitution in the proteoliposomes E, elution fraction; FT, flow through; W, wash.

Proteoliposomes (160 µl; 5 mg/ml) were treated with or without 2% DDM and incubated with 100 µl of Ni-Affinity Resin (Ni-Sepharose High Performance; GE Healthcare, Chicago, IL, USA) at 4°C for 1 h. The supernatant was collected, and the resin was washed 4 times using 50 µl of NA buffer (100 mM potassium phosphate pH 7, 300 mM KCl). The proteoliposomes were eluted in NA buffer containing 500 mM imidazole. Fifteen microliters of each fraction was mixed with 5 µl of loading blue buffer and analyzed by SDS-PAGE (**Fig. 1** and Supplemental Fig. S1). As a control, we next measured AmtB activity with the purified proteoliposomes containing only right-side-out (RSO)-inserted AmtB (Supplemental Fig. S2). The decay constant in the RSO-purified proteoliposomes or RSO/inside-out (IO) mixture was the same; hence, we concluded that it was justified not to purify the proteoliposomes before each SSME measurement.

### Sensor preparation for SSME measurement

Three millimeter gold-coated sensors (Nanion Technologies, Munich, Germany) were prepared as previously described (26). Briefly, 50 µl of a 0.5 mM octadecanethiol solution prepared in isopropanol was used to coat a thiol layer on the gold surface of the sensor during 30 min. The sensors were rinsed with isopropanol and deionized water, dried, and subsequently, diphytanoyl-*sn*-glycerol-3-phosphocholin solution was dropped onto the surface. One hundred microliters of NA buffer was immediately added to the sensor to form the solid supported membrane (SSM). Proteoliposomes/empty liposomes were defrosted and sonicated in a sonication bath (U300H Ultrawave Precision Ultrasonic Cleaning; Ultrawave, Cardiff, South Wales, United Kingdom) at 35 W for 1 min, diluted 10 times in NA buffer, and 10 µl was added at the surface of the SSM on the sensor. After centrifugation, the sensors were stored at 4°C for a maximum of 48 h before electrophysiological measurements.

### SSME measurements

The measurements were performed with a SURFE^2^R N1 machine (Nanion Technologies, Munich, Germany) using default parameters (27). The quality of the sensor was assessed before any recording by determining its capacitance (value should be between 15 and 30 nF) and conductance (value should be <5 nS). For the measurement, a single-solution exchange was used, which consisted of 3 phases of 1 s each; during which, NA, activating (A) (100 mM potassium phosphate pH 7, 300-X mM KCl, X mM of substrate -NH_4_^+^, MeA, Na^+^, K^+^-), and NA buffers were sequentially injected on the sensor over constant osmolality at a flow rate of 200 μl/s. The sample rate was set to 1000 Hz, and all the currents in the figures are presented at that rate, without filtering. The currents were amplified with a gain set to 10^9^ V/A.

Wherever stated, the raw transient curves were normalized against the maximum current and for the kinetics against the maximum current recorded after a substrate pulse of 100 mM.

The measurements were conducted ≥2 times on each sensor and on 6 sensors prepared from 2 independent batches of AmtB purification. The decay constant was fitted using Origin (OriginLab, Northampton, MA, USA), and the kinetic analysis was performed using Prism 7 (GraphPad Software, La Jolla, CA, USA). The current reconstruction was performed as previously described (26).

### Molecular dynamics simulations

The AmtB crystal structure at 1.35 Å resolution (PDB ID, 1U7G) (28) was used for all of our molecular dynamics simulations. The CHARMM-GUI web server graphical user interface (*http://www.charmm-gui.org/*) (29, 30) was applied to revert the mutations (S126P, K255L, F68S) present in the crystallographic construct in the PDB structure 1U7G back to its wild-type form. The protein termini were capped with acetyl and *N*-methyl moieties for the N and C terminus, respectively. The protein was then inserted into a 13 × 13-nm membrane patch constructed with the CHARMM-GUI web server interface. Three different membrane compositions were used: 1 containing 1-palmitoyl-2-oleoyl phosphatidic acid (POPA) and 1-palmitoyl-2-oleoyl phosphatidylcholine (POPC) lipids (POPA-to-POPC ratio, 1:9), and 2 containing POPA, POPC, and POPG lipids (POPA:POPC:POPG ratios, 1:9:2 and 1:9:10). K^+^ and Cl^−^ ions were added to neutralize the system and to obtain a bulk ionic concentration of 150 mM. The CHARMM36 force field was used for the protein, lipids, and ions (31, 32). The water molecules were modeled with the TIP3P water model (33). Water bonds and distances were constrained by the Settle method (34), and all other bonds by the LINCS method (35). After a steepest-descent minimization, the system was equilibrated for ∼5 ns by 6 consecutive equilibration steps (time ratio, 1:1:1:4:4:12) with decreasing position restraints on heavy atoms, ranging from 1000 to 200 kJ/mol/nm^2^. We thereby followed a protocol recommended by the CHARMM_GUI web server to construct and equilibrate mixed lipid bilayer systems (36). The first 3 equilibration steps were performed in a NVT ensemble (constant temperature/constant volume ensemble) using a Berendsen thermostat (37) to keep the temperature at 310 K. The subsequent steps were conducted under a NPT ensemble (constant temperature/constant pressure ensemble), switching on a Berendsen barostat (37) with isotropic coupling, to keep the pressure at 1 bar. Production molecular dynamics simulations were performed with a Nosé–Hoover thermostat (38) with a time constant of 0.2 ps, and a Parrinello–Rahman barostat (39, 40) with semi-isotropic pressure coupling. An integration time step of 2 fs was used throughout the simulations. All simulations were performed with the Gromacs software, v.5.11 (*http://www.gromacs.org/*) (41). The Visual Molecular Dynamics (VMD) software (*https://www.ks.uiuc.edu/Research/vmd/*) (42) was used for the visualization of the trajectories and the generation of all structural images. We used the VMD VolMap plugin for the generation of the volumetric density maps, the DSSP program (43, 44) for secondary structure assignment, and in-house Python code for additional trajectory analysis.

## RESULTS

### Characterization of AmtB activity by SSME

To measure ammonium transport activity by SSME, we purified AmtB as previously described (45). The protein was incorporated into liposomes containing a mixture of *E. coli* polar lipids/POPC at a weight ratio of 2:1. AmtB was reconstituted at an LPR of 10 w/w). DLS analysis confirmed that liposomes and proteoliposomes follow a unimodal size distribution with a mean size of 110 nm (Fig. 1*A*). To examine the orientation of AmtB inserted in the liposomes, we analyzed the proteoliposomes by immobilized metal affinity chromatography (IMAC) (46). If all AmtB proteins are inserted in an RSO orientation in a liposome, none of the C-terminal affinity tags should be accessible; hence, the proteoliposome should flow through the IMAC matrix. By contrast, if the AmtB protein is inserted IO in the proteoliposome, then the His-tag should be accessible, and the proteoliposome is expected to bind the matrix and thus be present in the elution fraction. As a control, we treated the proteoliposomes in parallel with DDM to solubilize AmtB and analyze it by IMAC using conditions identical to those used for the analysis of the proteoliposomes without DDM. Our analysis showed that more than half of the proteoliposomes are present in the flow through, demonstrating that, in >50% of the proteoliposomes, not a single protein is IO oriented, which signifies that most AmtB is orientated RSO in the proteoliposomes (Fig. 1*B*).

**Figure 2:**
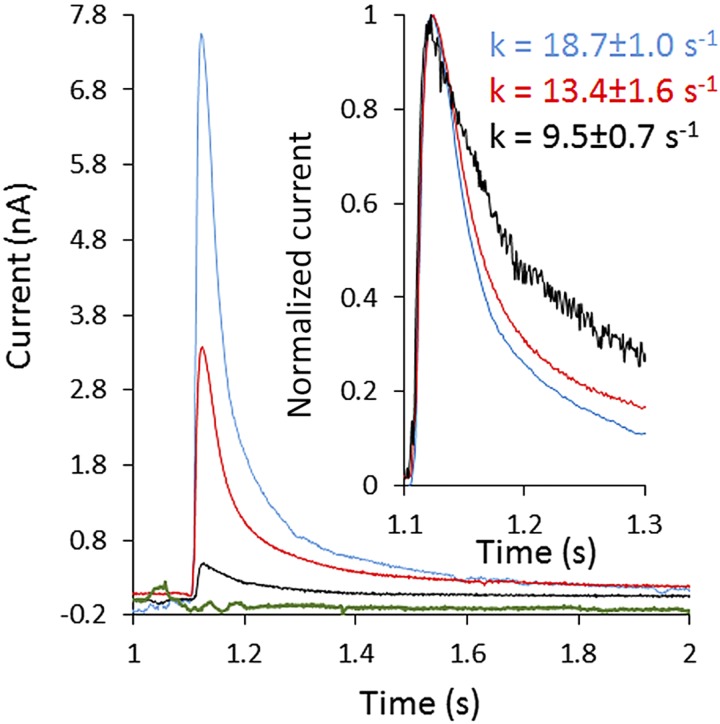
Characterization of AmtB activity. Transient current measured after a 100 mM ammonium pulse in empty liposomes (green) or proteoliposomes containing AmtB at an LPR of 50 (black), 10 (red), or 5 (blue). Inset: normalized current measured in proteoliposomes containing AmtB at an LPR of 50 (black), 10 (red), or 5 (blue).

**Figure 3:**
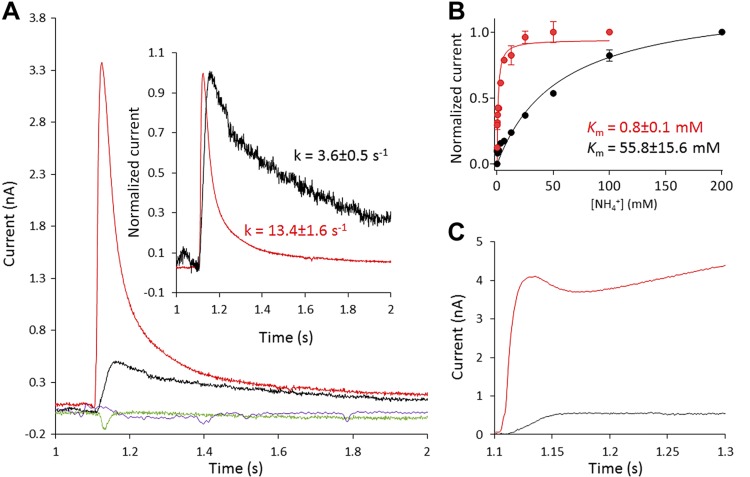
Specificity of AmtB activity. *A*) Transient current measured on proteoliposomes containing AmtB at LPR 10 after a 100 mM substrate pulse. Ammonium (red), methylammonium (black), potassium (purple), or sodium (green). Inset: normalized current after a 100-mM substrate jump. Ammonium (red), methylammonium (black). *B*) Substrate dependence [ammonium (red) or methylammonium (black)] of the maximum amplitude of the transient current. *C*) Reconstructed current using circuit analysis after a 100-mM pulse of ammonium (red) or methylammonium (black). SSME measures both presteady-state charge displacement (which corresponds to the binding of ammonium/MeA to AmtB) and steady-state charge displacement (which describes the continuous turnover of charge during the complete transport cycle of AmtB). It is possible, however, to isolate the steady-state (transport rate) current by analyzing the SSME system as an electric circuit describing the electrical properties of the compound membrane formed from the liposomes and the underlying SSM. This is important to clearly demonstrate that the rate of ammonium transport is larger than in the case of MeA.

SSME analysis after an ammonium pulse of 100 mM revealed a fast, positive, transient current of 3.3 nA in proteoliposomes, whereas no current was recorded for protein-free liposomes (**Fig. 2**). We show a representative trace in Fig. 2; however, the amplitude of the transient current differs from sensor to sensor because the number of proteoliposomes coated on the SSM varies (27). In our standard experimental setup, we measured the current 2 times on 6 sensors produced from 2 independent protein preparations. The average transient current peak measured for a pulse of 100 mM ammonium at LPR10 was 3.37 ± 0.26 nA. SSME records transient currents, because the charge displacement caused by the translocation of ammonium inside the proteoliposomes creates an outwardly directed negative membrane potential that progressively inhibits the transport cycle. This fast, transient current measures both presteady-state charge displacement (corresponding to the interaction of ammonium with AmtB) and steady-state charge displacement (describing the continuous turnover during the complete transport cycle of AmtB) (27). To further confirm that the transient currents correspond to the translocation of ammonium into the proteoliposomes, rather than a simple interaction between the substrate and the transporters, we investigated the effect of varying the number of transporters/proteoliposome on the transient current. It was expected that the decay time would be prolonged with increasing protein in the liposomes if the current represents a complete transport cycle, whereas it should be independent of the number of transporters/liposome if the current reflects a simple binding interaction between the substrate and the protein (47). To test that, we reconstituted AmtB into liposomes at LPR values of 50, 10, and 5 (w/w). After a pulse of 100 mM ammonium, the maximum amplitude of the transient current between LPR 50, 10, and 5 increased from 0.47 ± 0.02 to 3.37 ± 0.26 nA and 7.90 ± 0.35 nA, respectively. Importantly, the decay rate constant of the second phase increased from 9.5 ± 0.7 to 13.4 ± 1.6/s and 18.7 ± 1/s (Fig. 2). Taken together, these results show that a charge displacement specific to AmtB can be detected, and that the current describes the continuous turnover of the complete transport cycle. To determine the transport kinetics, the transient currents were measured in proteoliposomes reconstituted at LPR10, after ammonium pulses ranging from 0.024 to 100 mM. The peak currents saturated between ammonium pulses of 25–50 mM; therefore, we normalized our recordings against the current measured at 100 mM. The data were then fitted according to the Michaelis–Menten equation and a *K*_m_ of 0.8 ± 0.1 mM was calculated (*r*^2^ = 0.99) (**Fig. 3*B***).

To characterize the specificity of AmtB among monovalent cations, we measured the current after pulses of Na^+^ and K^+^. The ionic radii of Na^+^ (0.116 nm), and K^+^ (0.152 nm) are similar to the size of NH_4_^+^ ions (0.151 nm) (48). In spite of that, 100 mM Na^+^ or K^+^ pulses did not trigger any charge displacement (Fig. 3*A*). This shows that those ions do not interact with AmtB and are not translocated through the protein. These experimental observations agree with previous free-energy calculations, which have suggested a high energy barrier for the translocation of ions through AmtB because of the hydrophobicity of the pore (49–51).

Next, we investigated the specificity of AmtB for ammonium *vs.* methylammonium (MeA) transport. MeA has been widely used to measure ammonium transport activity because the radioactive tracer [^14^C]MeA is commercially available. However, the suitability of MeA as an ammonium analog to characterize the kinetics, specificity, and energetics of Amt/Mep/Rh protein activity has been questioned (52, 53). Here, we show that a pulse of 100 mM MeA triggers a transient current of 0.50 ± 0.02 nA, compared with 3.37 ± 0.26 nA for ammonium, whereas the decay constant is 4 times lower (Fig. 3*A*). The currents recorded by SSME are intrinsically transient; however, the transporter steady-state components can be reconstructed by circuit analysis (54). Steady-state transport in AmtB associated with 100 mM ammonium caused a current of ∼4 nA, whereas, for 100 mM MeA, it was ∼0.5 nA. This shows that MeA is translocated through AmtB at a greatly reduced rate compared with ammonium (Fig. 3*C*). Furthermore, kinetic analysis reveals a *K*_m_ 70 times higher (55.8 mM) for MeA compared with ammonium, showing further that MeA is a poor substrate analog for AmtB, not well suited to elucidate the mechanistic details of AmtB activity (Fig. 3*B*).

### POPG lipids are functionally required for full AmtB transport activity

**Figure 4:**
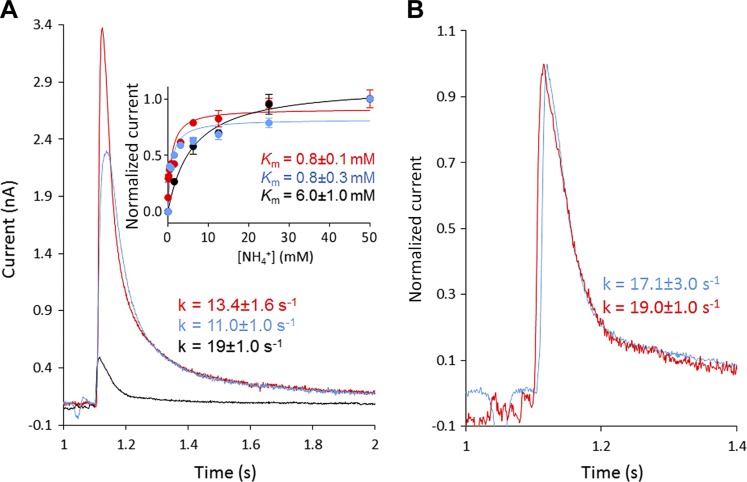
POPG is required for the full function of AmtB. *A*) Transient current measured after a 100-mM ammonium pulse in proteoliposomes containing the lipid conditions 2 (black), 3 (blue), and 1 (red). Inset: ammonium dependence of the maximum amplitude of the transient current for proteoliposomes containing the lipid conditions 2 (black), 3 (blue), and 1 (red). *B*) Normalized transient current measured in AmtB-containing proteoliposomes that do not contain POPG (condition 2) at LPR 10 (red) or 5 (blue).

Given that POPG has been shown to bind specifically to AmtB (23), we hypothesized that the lipid environment may also affect the protein’s activity, the ammonium–AmtB interaction, and/or the translocation process. To test that question, we reconstituted AmtB into liposomes containing a mixture of POPA/POPC at a weight ratio of 1:9 or ternary mixtures of POPA/POPC and POPG (**Table 1**). The POPA/POPC mixtures were chosen because no strong interactions have been detected between POPA, POPC, and AmtB (23), and also because AmtB is correctly inserted in this lipids mixture but does not translocate ammonium (the decay rate does not change at various LPRs; **Fig. 4*B***). Hence, the POPA/POPC lipid mixture is very well suited to assess the specific role of POPG in AmtB activity. The ternary mixture was chosen such that the quantity of POPG (16.5% w/w) matched the standard composition used for the previous experiments (*E. coli* polar lipids/POPC; 2/1 w/w) (Table 1). DLS measurements and IMAC analysis showed that the size of the liposomes, the protein orientation inside the membrane, and the quantity of protein inserted were equivalent for all lipid conditions (Supplemental Fig. S1*A*, *B*). In the absence of POPG (condition 2, Table 1), a 100-mM ammonium pulse triggered a transient current of 0.42 ± 0.04 nA, compared with 3.37 ± 0.26 nA in the presence of POPG (condition 1, Table 1) (Fig. 4*A*). Remarkably, in the presence of 16.5% POPG in an otherwise pure POPA/POPC condition (condition 3, Table 1), a current of 2.24 ± 0.06 nA was measured, which is >5 times greater than in the absence of POPG (condition 2, Table 1) (Fig. 4*A*). The decay and the *K*_m_ constants measured in the presence of POPG (conditions 1 and 3, Table 1) were similar and remained within the experimental error (Fig. 4). In contrast, in the absence of POPG (condition 2, Table 1), the decay rate and the *K*_m_ increased by 1.6- and 7-fold, respectively, compared with conditions 1 and 3 (Fig. 4*A*). All of these findings clearly show that POPG lipids are important for the transport activity of AmtB.

**Table 1.** Lipid composition in liposomes and total PG content^a^

Lipid condition	Lipid content-ratio (w/w)	% PG
1	*E. coli* polar/PC - 2/1	16.5
2	PA/PC - 1/9	0
3	PA-PC/PG - 5/1	16.5

a We reconstituted AmtB in liposomes containing a mixture of phosphatidic acid (PA)/phosphatidylcholine (PC) at a weight ratio of 1/9 (condition 2) or in PA/PC-containing liposomes from condition 2 but also containing PG at a weight ratio of 5/1 (condition 3). Condition 3 was chosen such that the quantity of PG (16.5% w/w) matched the standard composition used for the previous experiments (*E. coli* polar lipids/PC 2/1 w/w; condition 1).

To determine whether AmtB completes a full transport cycle in the absence of POPG, we reconstituted the protein in condition 2 (without POPG, Table 1) at varying LPRs of 50, 10, and 5 (w/w). By SDS-PAGE analysis, we carefully checked that the quantity of protein in conditions 1 and 2 at the 3 LPRs were comparable (Supplemental Fig. S1*C*). Under condition 2, an ammonium pulse of 100 mM did not trigger a measurable transient current at LPR 50; however, at LPR 10 and 5, the decay time was similar and within the experimental error (19.0 ± 1.0/s *vs.* 17.1 ± 3.0/s respectively; Fig. 4*B*).

**Figure 5:**
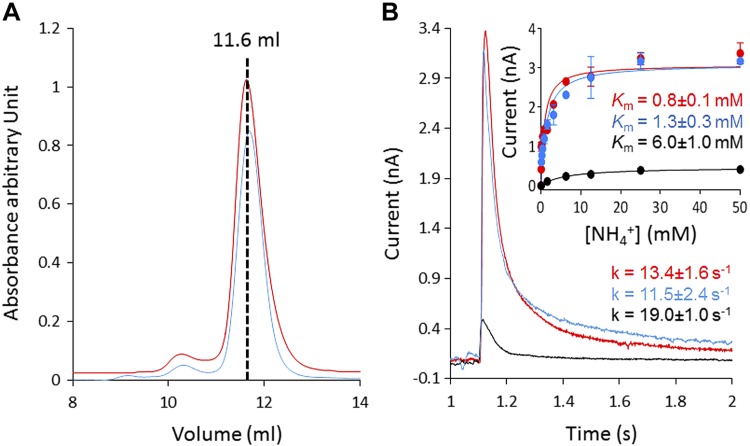
AmtB is correctly folded in the absence of POPG. *A*) Gel filtration trace (Superdex 200 10/300 increase) of AmtB before (red) insertion in proteoliposomes under condition 2 and after (blue) solubilization from proteoliposomes under lipid condition 2 (Table 1). *B*) Transient current measured after a 100 mM ammonium jump in proteoliposomes under condition 2 (black), under condition 1 containing AmtB reinserted after solubilization from proteoliposomes under condition 2 (blue), and under condition 1 (red) (Table 1). Inset: ammonium dependence (raw data) of the maximum amplitude of the transient current in proteoliposomes under condition 1 (red), 2 (black), or 4 (blue) (Table 1).

To ensure that AmtB was not misfolded in the liposomes under condition 2 (without POPG, Table 1), we solubilized the proteoliposomes in 2% DDM to extract AmtB and analyzed the protein by SEC on a Superdex 10/300 increase column (GE Healthcare). The SEC analysis showed that the protein elutes as a single monodispersed peak at the same retention time (11.6 ml) as before reconstitution under condition 2 (**Fig. 5*A***). We subsequently reinserted AmtB solubilized from condition 2 (without POPG, Table 1) into proteoliposomes using condition 1 (with POPG, Table 1). An ammonium pulse of 100 mM in these proteoliposomes triggered a transient current of 3.18 ± 0.09 nA with a decay constant of 11.5 ± 2.4/s, and the kinetic analysis revealed a *K*_m_ of 1.3 ± 0.3 mM (Fig. 5*B*). These results show that AmtB reconstituted under condition 2 (without POPG) regains the original activity parameters (maximum current intensity, decay time, and *K*_m_) when rereconstituted in liposomes under condition 1 (with POPG). These data confirm correct folding of AmtB in the proteoliposomes without POPG (condition 2). Taken together, these findings show that POPG is important for AmtB activity and furthermore indicate that, in the absence of POPG, AmtB exhibits a defective transport cycle.

### Structural and dynamic investigation of POPG interacting with AmtB

**Figure 6:**
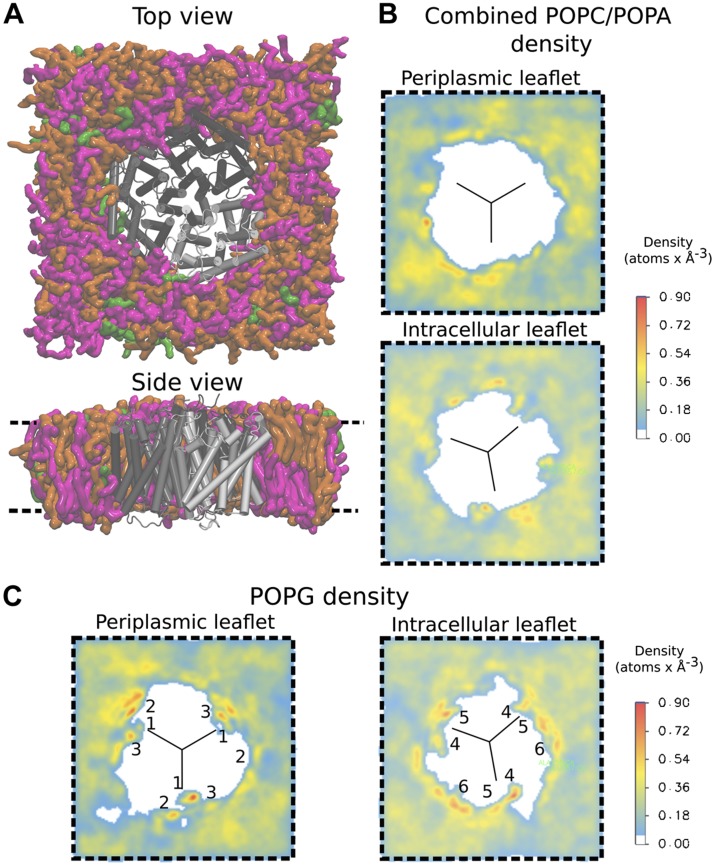
Trimeric AmtB in the POPA/POPC/POPG (1:9:10) system and lipid density plots. *A*) Final frame of the simulation system, seen from the periplasm (top) and from the side (bottom). The protein is shown in gray, the POPC lipid molecules in orange, POPA in green, and POPG in magenta. *B*) Volumetric analysis of the POPC and POPA combined average densities over the entire 700-ns trajectory. *C*) Volumetric analysis of POPG average densities over the entire trajectory. The black lines in the density plots mark the approximate monomer interfaces. Specific binding sites are labeled in the density plots. Comparison of the 2-dimensional density maps shows that POPG tends to localize preferentially close to the monomer interfaces.

We next applied atomistic MD simulations to study the interaction of POPG lipids with AmtB in membranes on the molecular level. **Figure 6*A*** and Supplemental Figs. S3*A* and S4*A* show the simulation systems containing an AmtB trimer embedded within POPA/POPC/POPG (1:9:10), POPA/POPC (1:9), and POPA/POPC/POPG (1:9:2) mixed lipid membranes, respectively. The color maps on the right and bottom of each of these figures display the density of POPA/POPC and POPG, respectively, each derived from 0.7-μs simulations. The slices representing the lipid density in the periplasmic and cytoplasmic leaflets were taken at the average *z* axis position of the phosphorus atoms within the lipid head groups of each membrane leaflet. The AmtB trimer remains stable in all the lipid environments we studied (Supplemental Fig. S5).

**Figure 7:**
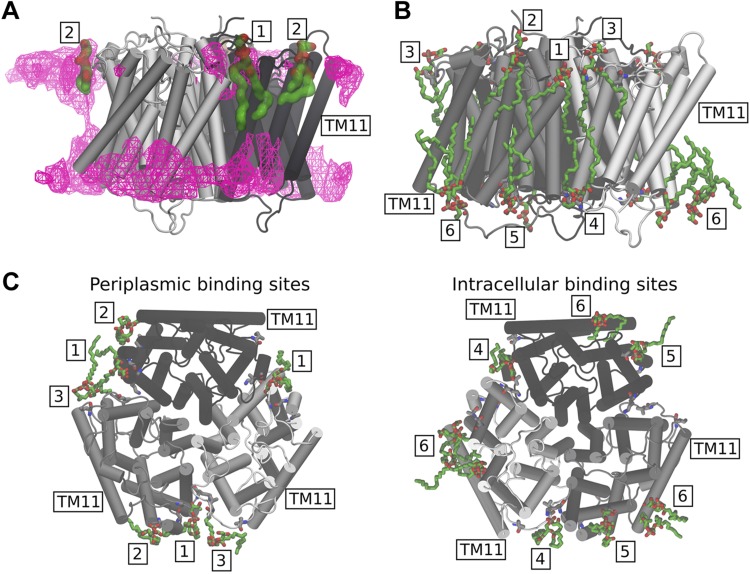
The images highlight the POPG binding locations observed in the POPA/POPC/POPG (1:9:10) simulations. The binding sites located in the periplasmic leaflet are numbered 1, 2, and 3, whereas the intracellular ones are labeled 4, 5, and 6. The same numbering scheme has been adopted for the volumetric maps shown in Supplemental Figs. S2 and S3. *A*) Volumetric map of the average POPG density obtained from a 700-ns simulation (magenta mesh surface, isovalue = 0.38) is compared with the POPG binding sites, which were previously resolved in the X-ray structure (PDB ID, 4nh2; lipids in green). Generally, good agreement between the experimental and the simulation sites was observed, especially for binding site 1. *B*, *C*) Side (*B*), periplasmic (*C*, left), and intracellular (*C*, right) views of the POPG binding sites taken from a representative simulation frame from the POPA/POPC/POPG (1:9:10) mixture. Generally, POPG tends to bind close to the monomer interface.

Although specific AmtB interactions with POPG had previously been detected crystallographically for the extracellular membrane leaflet (23), our simulations revealed additional POPG binding sites on the intracellular side of the membrane (Fig. 6*B*, *C*). POPG lipids preferably occupy sites on the AmtB trimer located near the interfaces between the monomers, both within the periplasmic and intracellular membrane leaflets (Fig. 6*C*). Specifically, we observe 3 high-density lipid interaction sites/subunit in the periplasmic leaflet, 1 at the monomer interface (site 1) and 2 in its vicinity (sites 2 and 3). Within the intracellular leaflet, about 2 high-density lipid sites are seen per subunit, in which lipids interact with interfacial helices of AmtB in both cases (Fig. 6*C*; sites 4 and 5), and an additional high-density region, in which we observed POPG clustering (site 6). By contrast, the density maps recorded for the POPA/POPC lipid mixture show no binding hotspots of AmtB for POPA and POPC within the periplasmic membrane leaflet, whereas some lipid accumulation was observed within the intracellular leaflet close to the AmtB POPG binding sites 1 and 2 (Supplemental Fig. S3). The POPG density maps obtained from the POPA/POPC/POPG (1:9:2) simulation (Supplemental Fig. S4*C*) showed that, because of the lower POPG concentration in this simulation system, the binding regions observed in the POPA/POPC/POPG (1:9:10) simulation were only partially occupied by POPG. Close-up images highlighting the interaction sites between POPG lipids and the AmtB subunits are shown in **Fig. 7**. The binding sites observed in our MD simulations within the periplasmic leaflet are in good agreement with crystallographically defined sites (23) (Fig. 7*A*), whereas the new intracellular interaction sites also located to the interface region between the AmtB subunits (Fig. 7*B*, *C*).

Supplemental Figure S6 shows the time evolution of the occupancy of all the suggested AmtB lipid binding sites by each lipid type, using data from 2 independent simulations of AmtB in a POPA/POPC/POPG (1:9:10) membrane. As can be seen, the sites we identified preferably interact with POPG lipids. In addition, an analysis of the number of hydrogen bonds formed between the lipid head groups and the protein (Supplemental Fig. S7*A*), as well as the radial distribution of the lipids around the protein (Supplemental Fig. S7*C*), shows that POPG tends to localize closer to the protein surface, compared with the POPA and POPC lipids, and to form more hydrogen bond contacts. The average number of hydrogen bonds among each protein residue and the lipid head groups is displayed in Supplemental Fig. S7*B*, confirming that POPG lipids generally establish more hydrogen bonds, in particular, near residues that form the reported binding sites (1–6 in Fig. 7).

The most conspicuous conformational change we found to be induced by POPG binding in AmtB on the time scale of our simulations was the preferred formation of a short helix within a periplasmic loop region (residues 77–81) after binding of the charged POPG head group. The propensity of that region to form an α helix is shown in Supplemental Fig. S8. The simulations containing POPG (both in the ratios 1:9:2 and 1:9:10) exhibited a significantly increased propensity for this loop region to form a helical structure compared with simulations in the absence of POPG. That short region is part of a loop, which Laganowsky *et al.* (23) showed to adopt a slightly different conformation upon POPG binding, compared with the unbound structure, albeit without formation of a helix. Although the detailed mechanism of ammonium transport in AmtB is still unclear, this finding might link POPG binding to AmtB structure and function.

## DISCUSSION

A plethora of functional studies aimed at elucidating the mechanism of ammonium transport by Amt proteins has led to considerable controversy because of the lack of an *in vitro* assay characterizing the activity of Amt proteins using ammonium as the substrate (1, 9). The elegant work of Wacker *et al*. (55) showed that 2 ammonium transporters from *Archaeoglobus* *fulgidus* activity are electrogenic (55). The work we present here shows that the activity of the archetypal Amt/Mep/Rh protein AmtB is also associated with charge translocation across the membrane, which suggests that electrogenic transport may be a general feature for these proteins. However, we also demonstrated that, contrary to *A. fulgidus* Amt1, AmtB highly discriminates between MeA and ammonium as substrates. Although in *A. fulgidus* Amt1, MeA triggers a transient current amounting to 87% of the current elicited by ammonium (55), the current induced by MeA in AmtB is <15% of that observed for ammonium. The structural basis for that discrepancy is not yet clear because the 3 principal conserved features, namely the S1 binding site, the “Phe gate,” and the pore twin–His motif are structurally similar between both proteins. However, that functional difference, at the molecular level, between eubacterial and archaeal Amts raises important questions about the universality of the transport mechanism in microbial ammonium transporters. This is particularly relevant in the case of the Mep2-like protein, which has been assigned a sensor role in filamentous development, often related to the virulence of pathogenic fungi (13). There are 2 major hypotheses concerning the molecular mechanism of Mep2-mediated signaling. The first is that Mep2 is a sensor interacting with signaling partners, leading to induction of filamentation (56, 57), whereas the other posits that the transport mechanism of Mep2-like proteins may differ from other Mep proteins (58–60). Current evidence favors the second hypothesis given that signaling efficiency is closely linked to transport efficiency; however, further studies are needed to elucidate the exact mechanism, which may provide important information for the design of novel antifungal therapies.

It is well established that lipids can affect membrane protein structure and function through bulk membrane effects by direct, but transient, annular interactions with the bilayer-exposed surface of the protein or by specific lipid binding to protein sites (for review, see Denning and Beckstein (61). Altogether 8 molecules of POPG have been resolved in a recent crystal structure of AmtB, interacting at specific binding sites within the extracellular membrane leaflet (23). Our MD simulations identify further interactions of POPG molecules in the inner leaflet of the membrane. However, so far, no functional relationships have been reported to link POPG binding with the activity of AmtB. Here, we show that, in the absence of POPG, AmtB is nonfunctional as a transporter and unable to complete the full translocation cycle. Our experiments and simulations do not indicate substantial conformational changes in the S1 periplasmic binding site or in the pore, suggesting that the molecular basis of the POPG effect on AmtB activity could involve novel mechanistic sites. In this context, it is interesting to compare our findings with the lactose permease LacY in *E. coli*, the most extensively studied secondary transporter in the context of lipid–protein interactions. In the absence of POPC and/or phosphatidylethanolamine (POPE), LacY is unable to support active transport of the substrate into the cell, although the binding of the substrate to the protein is unaffected (62). This is similar to the observations we made for AmtB in the absence of POPG lipids. LacY is known to undergo drastic topological rearrangements, which may explain the effect of POPC/POPE on its activity (63). In the case of AmtB, no topological rearrangements were observed upon POPG binding, and we have shown that, in the absence of POPG, AmtB was folded correctly in the proteoliposomes, such that a major change in the AmtB topology is unlikely to explain the functional role of POPG. We did, however, observe changes in the dynamics of AmtB subunits, which located particularly to loop regions at the periplasmic face of the protein. The periplasmic loop regions have been shown to include functionally important residues (49, 50, 64, 65). Once a more detailed picture of the overall transport mechanism of AmtB has been obtained, the role of those effects may emerge into greater clarity.

Furthermore, several lines of evidence point toward functional cooperativity between the 3 subunits in the Amt/Mep transporters. First, in *S. cerevisiae*, it has been demonstrated that expression of a nonfunctional Mep1 protein inhibits the transport activity of Mep2 and Mep3, indicating crosstalk between different Mep transporters (66), and similar observations have been reported for ammonium transporters of *Aspergillus nidulans* (67). Second, coexpressed, nonfunctional monomers cross-inhibit transport in plant Amts, and a genetic screen has identified several mutations at the subunit interface of the *A. thaliana* Amt1/2 transporter, which inactivated the translocation activity (8, 68). Third, extensive site-directed mutagenesis of the C-terminal tail of *E. coli* AmtB has led to the hypothesis that the 3 subunits function in a cooperative manner (69). All of these findings indicate functional coupling among the adjacent subunits. Previous structural data and our MD simulations showed that POPG molecules bind mainly to sites at the vicinity of the subunit interfaces. It is, therefore, attractive to hypothesize that POPG molecules act as wedges, which mediate the functional interaction between the subunits. In line with that hypothesis, a model in which various AmtB conformations may be favored upon specific lipid binding has been proposed (24). Finally, it has recently been shown that other lipids, including POPE and cardiolipin, can bind AmtB allosterically, indicating that transporters may recruit their own microlipidic environment (70). Whether these binding events are important to modulating the activity of AmtB remains a question to be addressed in future studies.

## Supplementary Material

This article includes supplemental data. Please visit *http://www.fasebj.org* to obtain this information.

## Abbreviations

A: activated
Amt: ammonium transporter
AmtB: *Escherichia coli* ammonium transporter
DDM: *n*-dodecyl-β-d-maltoside
DLS: dynamic light scattering
IMAC: immobilized metal affinity chromatography
IO: inside-out
LPR: lipid-to-protein ratio
MD: molecular dynamics
MeA: methylammonium
Mep: methylammonium permease
NA: nonactivated
PDB: Protein Data Bank
POPA: 1-palmitoyl-2-oleoyl phosphatidic acid
POPC: 1-palmitoyl-2-oleoyl phosphatidylcholine
POPE: 1-palmitoyl-2-oleoyl phosphatidylethanolamine
POPG: 1-palmitoyl-2-oleoyl phosphatidylglycerol
Rh: rhesus protein
RSO: right-side-out
SEC: size-exclusion chromatography
SSM: solid supported membrane
SSME: solid supported membrane electrophysiology
