# Supplementary material for: The lipid environment determines the activity of the Escherichia coli ammonium transporter AmtB
Source: FASEB J. 2018 Sep 13;33(2):1989–99. doi: 10.1096/fj.201800782R (PMC6338640; doi:10.1096/fj.201800782R)
Supplement: Supplementary file 1 [file fj.201800782R.sf1.pdf]

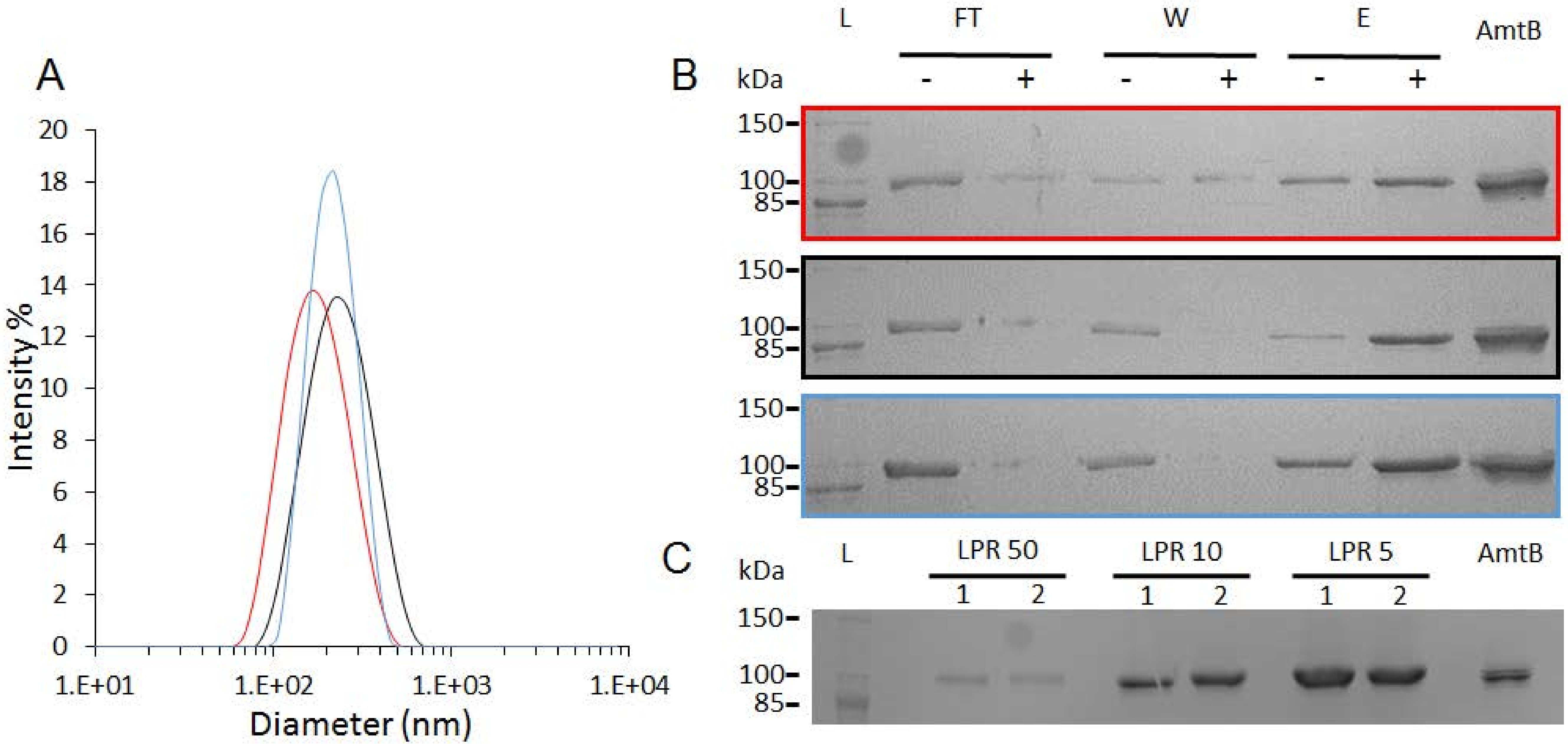
A
Intensity %
20
18
16
14
12
10
8
6
4
2
0
1.E+01
1.E+02
1.E+03
1.E+04
Diameter (nm)
B
kDa
L
FT
W
E
AmtB
-
+
150
100
85
C
LPR 50
LPR 10
LPR 5
1
2

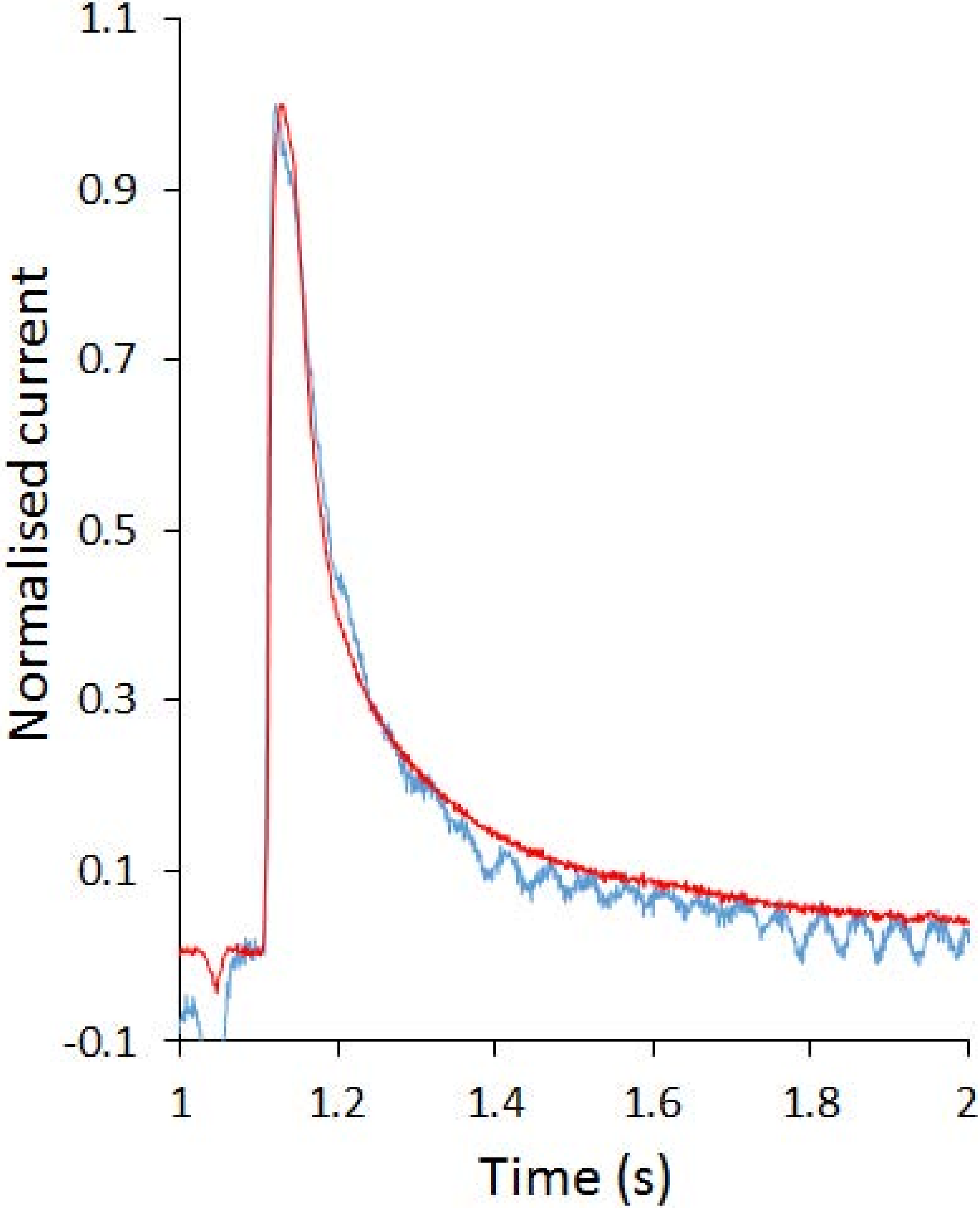
Normalised current
1.1
0.9
0.7
0.5
0.3
0.1
-0.1
1
1.2
1.4
1.6
1.8
2
Time (s)

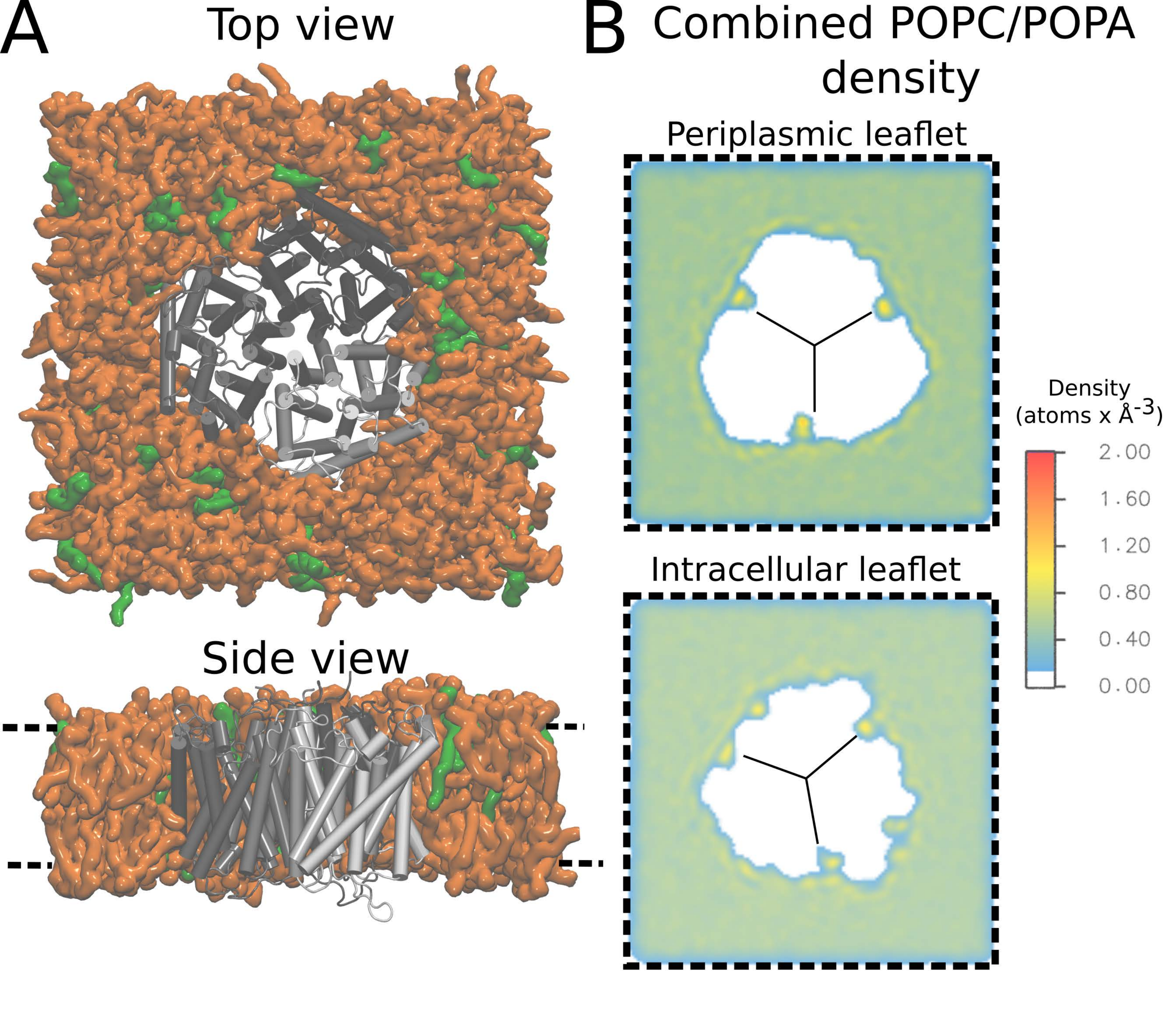
A
Top view
Side view
B
Combined POPC/POPA density
Periplasmic leaflet
Intracellular leaflet
Density (atoms x Å^{-3})
2.00
1.60
1.20
0.80
0.40
0.00

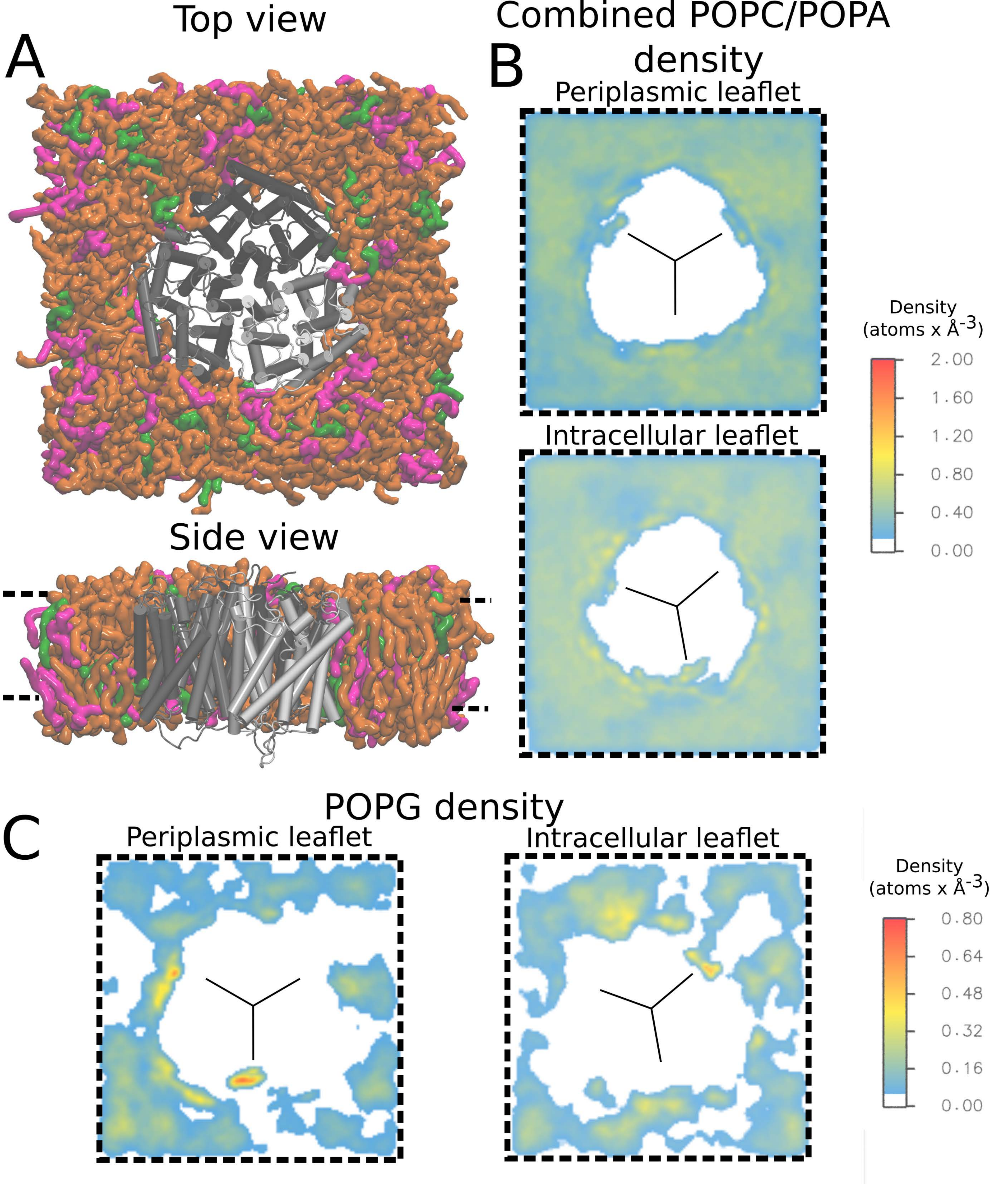
Top view
A
Combined POPC/POPA density
B
Periplasmic leaflet
Intracellular leaflet
Density (atoms x Å^{-3})
2.00
1.60
1.20
0.80
0.40
0.00
Side view
C
POPG density
Periplasmic leaflet
Intracellular leaflet
Density (atoms x Å^{-3})
0.80
0.64
0.48
0.32
0.16
0.00

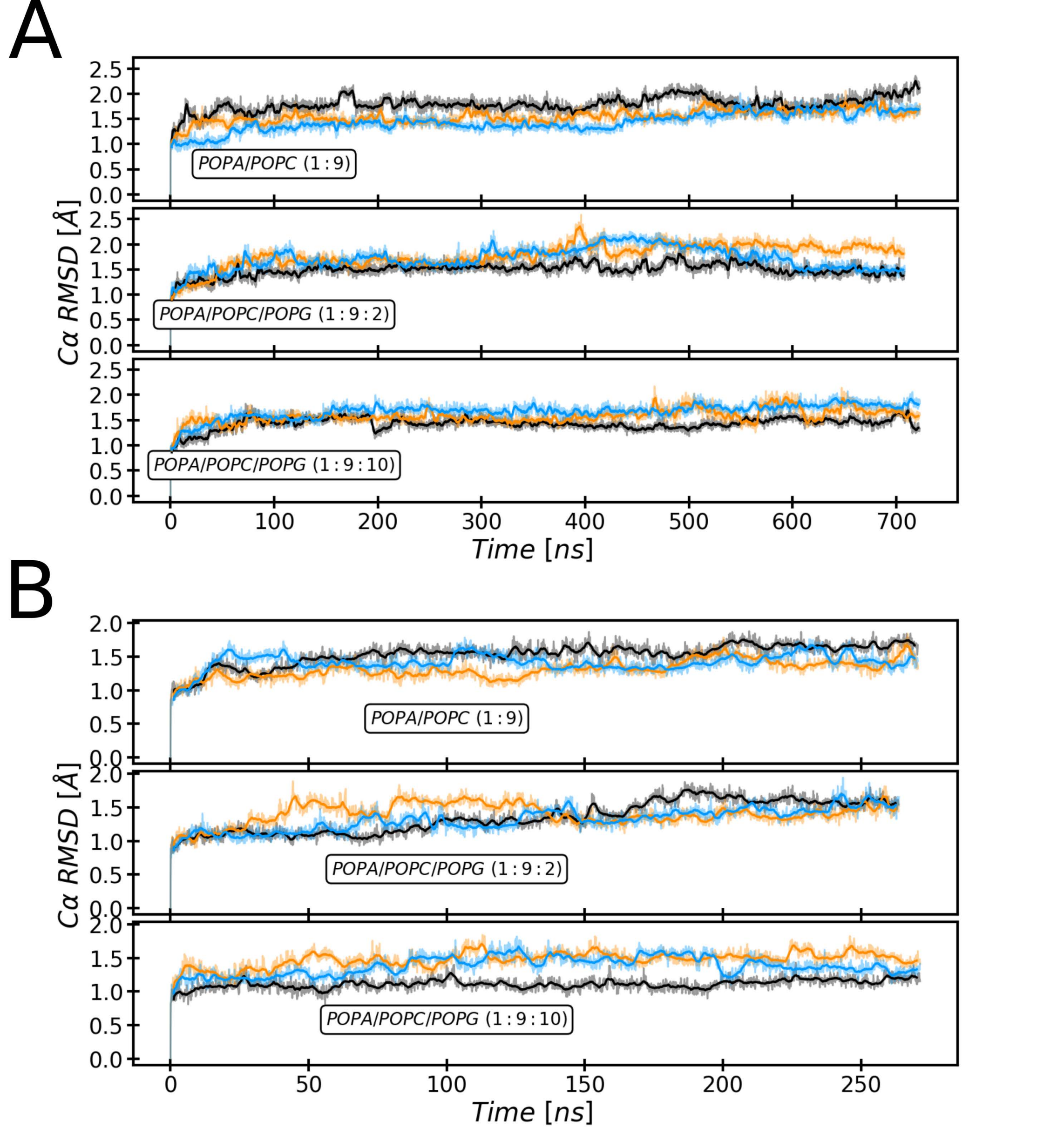

A
B
Cα RMSD [Å]
POPA/POPC (1 : 9)
POPA/POPC/POPG (1 : 9 : 2)
POPA/POPC/POPG (1 : 9 : 10)
Time [ns]

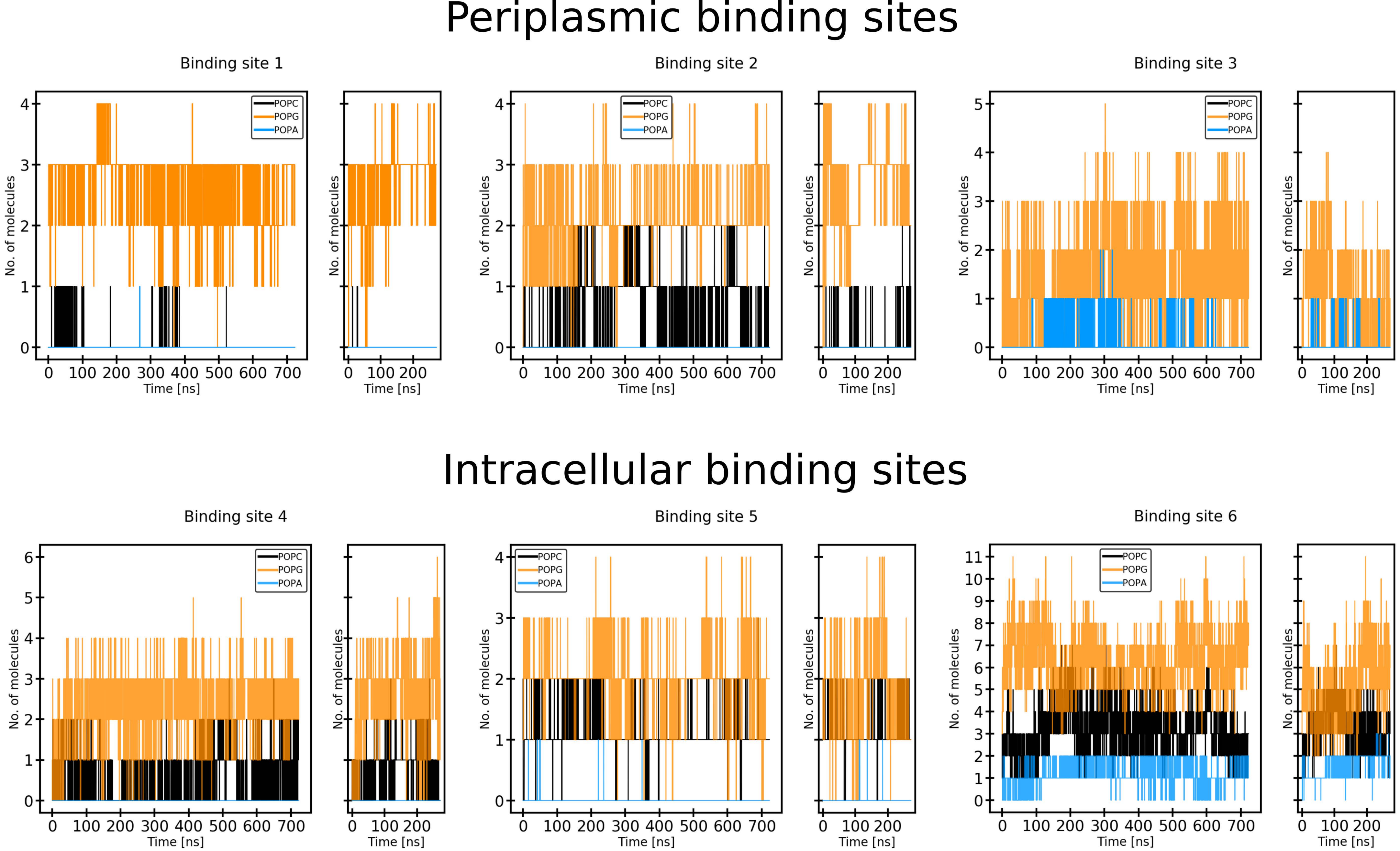

Periplasmic binding sites
Binding site 1
Binding site 2
Binding site 3
POPC
POPG
POPA
No. of molecules
Time [ns]
Intracellular binding sites
Binding site 4
Binding site 5
Binding site 6

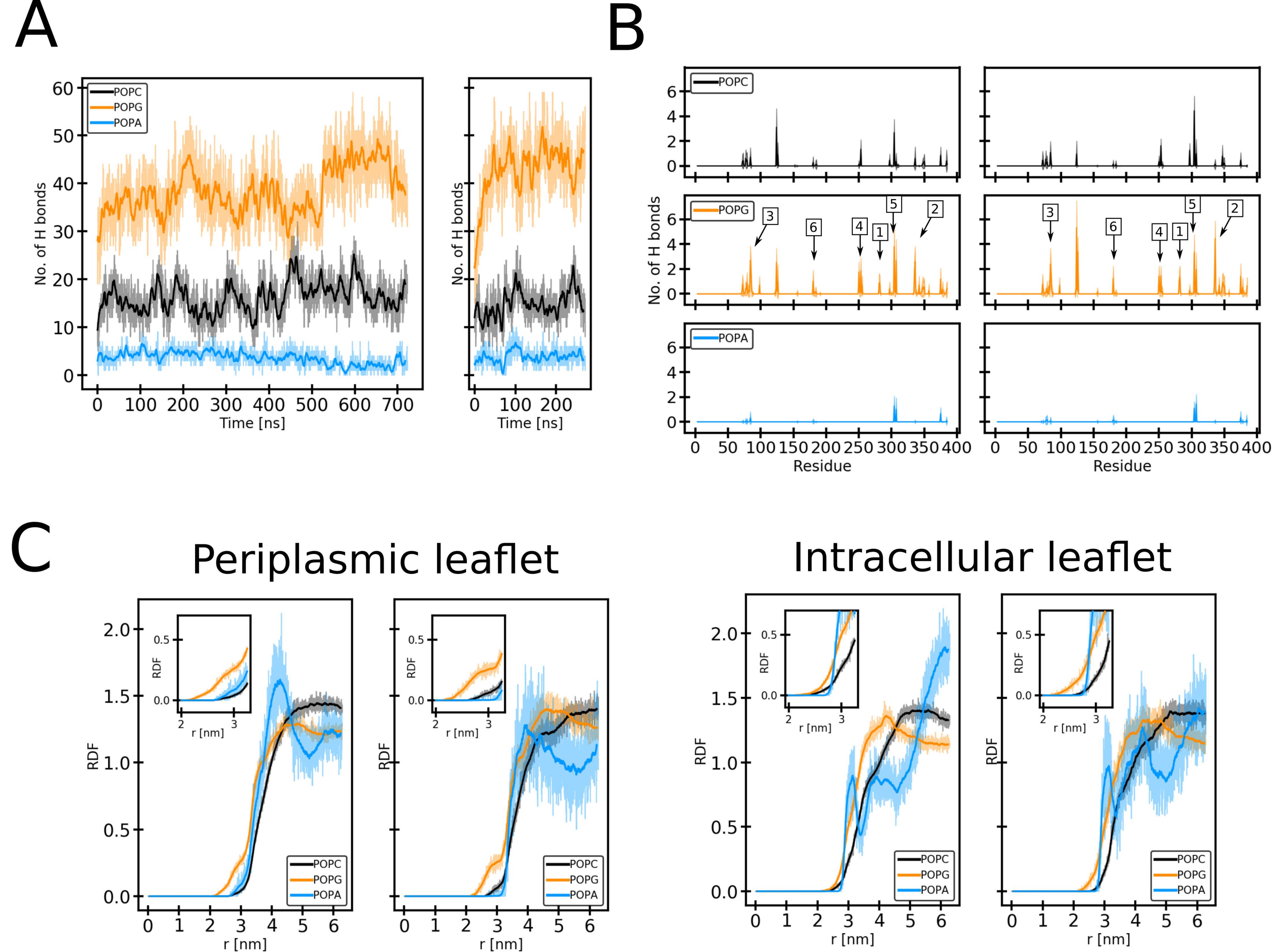

A
B
C
Periplasmic leaflet
Intracellular leaflet
POPC
POPG
POPA
No. of H bonds
Time [ns]
Residue
RDF
r [nm]

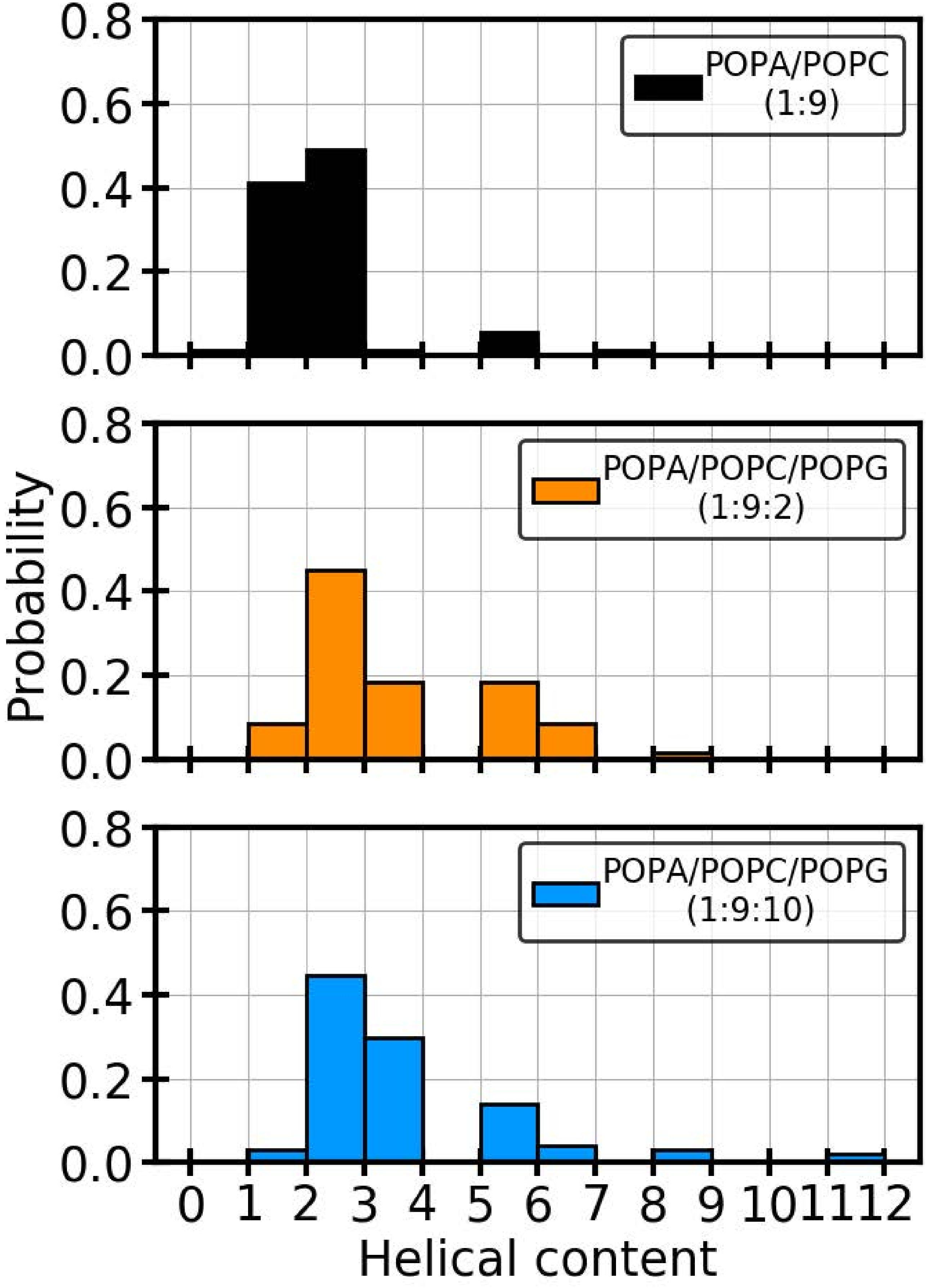

POPA/POPC (1:9)
POPA/POPC/POPG (1:9:2)
POPA/POPC/POPG (1:9:10)
Probability
Helical content
0.0
0.2
0.4
0.6
0.8
0 1 2 3 4 5 6 7 8 9 10 11 12

Legends supplementary figures

Figure S1: AmtB orientation and size in liposomes with different lipid composition. **(A)** DLS analysis of the proteoliposomes made using lipid condition 1 (red), 2 (black) or 3 (blue) at LPR 10. **(B)** SDS-PAGE Coomassie Blue-stained gel of the proteoliposomes made using lipid condition 1 (red square), 2 (black square) and 3 (blue square) purified by IMAC after DDM treatment (+) or in absence of DDM (-). FT; flow through, W; wash, E; elution fraction, AmtB; 5µg of pure AmtB used for the reconstitution in the proteoliposomes. **(C)** Comparison of the proteins content in proteoliposomes prepared under condition 1 and 2 (Table 1) at LPR 50, 10 and 5 (wt/wt). 10 µL of liposomes at 5mg/ml have been loaded on the gel. AmtB: 5µg of pure AmtB.

Figure S2: Characterization of AmtB activity in pure RSO v.s. mixture of IO/RSO proteoliposomes. Transient current measured after a 100 mM ammonium pulse in pure RSO (blue) or mixture of IO/RSO (red) proteolipososme.

Figure S3: Trimeric AmtB in the POPA/POPC (1:9) system and lipid density plots. **(A)** Last frame of the simulation system, viewed from the periplasm (top) and from the side (bottom). The protein is shown in grey, the PC molecules in orange, and the PA lipids in green. **(B)** Volumetric analysis of PC and PA average densities over the whole 700 ns trajectory, with a 100 ps frame step. Note the different colour scale with respect to main text Figure 6.

Figure S4: Trimeric AmtB in the POPA/POPC/POPG (1:9:2) system and lipid density plots. **(A)** Last frame of the simulation system, viewed from the periplasm (top) and from the side (bottom). The protein is shown in grey, the PC molecules in orange, and the PA lipids in green, and PG in magenta. **(B)** Volumetric analysis of PC and PA average densities over the whole 700 ns trajectory, with a 100 ps frame step. Note the different colour scale with respect to main text Figure 6.

Figure S5: Ca RMSD of AmtB during the simulations in various lipid mixtures. The RMSD remains well below 2.5 Å in all of the simulations. This shows that, regardless of the membrane composition, the protein is stable and maintains its overall conformation in the simulations. (A) displays the RMSD over 700-ns simulations; (B) shows the RMSD during independent 250-ns simulations.

Figure S6: Timeline of the occupancy of the POPG binding sites. Occupancy for the periplasmic leaflet (top) and the intracellular leaflet (bottom) with all lipids investigated. Preference for occupancy by POPG molecules is seen for all of the binding sites.

Figure S7: Interactions between the various lipid types and AmtB. **(A)** Timeline of the total number of hydrogen bonds made between the protein and each lipid type, shown for the POPA/POPC/POPG (1:9:10) simulations. Shaded lines show raw data, bold lines represent a running average using 5-ns windows. It can be seen that the POPG lipids quickly establish and maintain a higher number of H-bonding contacts with AmtB. **(B)** Average number of hydrogen bonds for each AmtB residue (standard deviation in shaded colours). The POPG binding sites observed in the simulations are labelled. **(C)** Radial distribution function (RDF) of each lipid for the periplasmic (left) and the intracellular (right) leaflets. Shaded lines show the raw data, bold lines represent a running average using 2.5-ns windows. The graphs show that the POPG molecules tend to localize closer to the protein surface, compared to the other lipids, in both leaflets (see especially insets, which display a focus of the RDF near the protein surface).

Figure S8: Helix-forming propensity of AmtB residues 77-81. Data from the two simulations conducted for each membrane lipid mixture was aggregated. The simulations containing POPG show a significantly raised tendency of this region to form a short alpha-helix (p-value$<10^{-7}$).
